# Patients with sporadic and familial amyotrophic lateral sclerosis found value in genetic testing

**DOI:** 10.1002/mgg3.360

**Published:** 2017-12-20

**Authors:** Karin N. Wagner, Haikady N. Nagaraja, Dawn C. Allain, Adam Quick, Stephen J. Kolb, Jennifer Roggenbuck

**Affiliations:** ^1^ Genetic Counseling Graduate Program The Ohio State University Columbus OH USA; ^2^ Division of Biostatistics The Ohio State University College of Public Health Columbus OH USA; ^3^ Division of Human Genetics The Ohio State University Wexner Medical Center Columbus OH USA; ^4^ Department of Neurology The Ohio State University Wexner Medical Center Columbus OH USA; ^5^ Department of Biological Chemistry and Pharmacy Wexner Medical Center The Ohio State University Columbus OH USA

**Keywords:** access to care, amyotrophic lateral sclerosis genetics, genetic counseling, genetic testing, patient attitudes

## Abstract

**Background:**

Amyotrophic lateral sclerosis (ALS) is increasingly recognized as a genetic disease. There is no consensus, however, as to the role of genetic testing in the care of the ALS patient.

**Methods:**

We conducted a survey to study patient access, attitudes, and experience with ALS genetic testing among patients enrolled in a US ALS registry.

**Results:**

Among 449 survey respondents, 156 (34.7%) were offered testing and 105 of 156 (67.3%) completed testing. The majority of respondents with familial ALS (fALS) (31/45, 68.9%) were offered testing, while a minority of respondents with sporadic ALS (sALS) (111/404, 27.5%) were offered testing (*p* = .00001). Comparison of mean test experience scores between groups revealed that respondents with fALS were no more likely to report a favorable experience with genetic testing than those with sALS (*p* = .51). Respondents who saw a genetic counselor did not have significantly different test experience scores, compared to those who did not (*p* = .14). In addition, no differences in test experience scores were observed between those who received positive or negative genetic test results (*p* = .98).

**Conclusion:**

These data indicate that patients with ALS found value in clinical genetic testing.

## 1 INTRODUCTION

Amyotrophic lateral sclerosis (ALS) is increasingly recognized as a genetic disease. The genetic basis of ~70% of familial ALS (fALS) and ~10% of sporadic ALS (sALS) is now understood (Renton, Chio, & Traynor, 2014). Emerging data suggest that the proportion of sALS patients carrying a highly penetrant mutation may be even greater in the context of a family history of dementia (Umoh et al., 2016). Commercial genetic testing for ALS is now widely available, in the form of assays for the *C9orf72* (OMIM: 614260) repeat expansion, single‐gene sequencing, large multigene sequencing panels, and exome sequencing. However, there is no consensus as to the role of genetic testing in the care of the ALS patient, and US practice guidelines do not provide recommendations for the offer of genetic testing (Miller et al., 2009).

Several national and international studies have examined ALS genetic testing practices among neurologists. In an international survey, Byrne, Elamin, Bede, and Hardiman (2012) found that 67.0% of neurologists offered genetic testing to fALS patients, and 10.3% offered testing to sALS patients. Arthur et al. (2016) surveyed US neurologists and reported that 93.0% offer testing to fALS and 30.2% to sALS patients. Most recently, Vajda et al. (2017) reported that 90.2% and 49.4% of international neurologists offer testing to fALS and sALS patients, respectively. These studies reveal that, in the last 5 years, an increasing percentage of neurologists are offering ALS genetic testing in clinical practice. However, patient outcomes related to ALS genetic testing have not been studied, and as therapeutic interventions for genetic forms of ALS are not available outside of clinical trials, it is not known whether patients find value in genetic testing.

In order to study patient access, attitudes, and experience with ALS genetic testing, we conducted a survey of ALS patients enrolled in a national ALS registry. Data collected on respondent demographics, understanding of ALS genetics, and attitudes toward genetic testing were previously reported (Wagner et al., 2016). Here, we report data collected from a subset of respondents who actually underwent genetic testing. We examine respondent experience with the genetic testing process, investigate factors potentially associated with a positive testing experience, and make suggestions for clinical practice.

## 2 MATERIALS AND METHODS

### 2.1 Ethical compliance

This survey was conducted with approval from the Institutional Review Board (IRB) from The Ohio State University Wexner Medical Center, Columbus, Ohio. Specific, separate ethics committee review and/or approval for this study was not indicated per the IRB review.

Study subjects completed an anonymous online survey using the survey engine SurveyMonkey^®^. Patients affected with ALS were eligible to participate; caregivers were permitted to assist, if needed. One survey was completed per respondent. Eligible participants were identified and contacted through the United States Centers for Disease Control–Agency for Toxic Substance and Disease Registry (CDC–ATSDR). The CDC–ATSDR advertised and distributed the survey link via a one‐time email announcement, after approval from the ATSDR Committee. All results were tabulated and exported in .xls and/or .csv format for analysis.

The survey instrument was described in detail previously (Wagner et al., 2016), and is available in Supporting Information. Those respondents who were offered and had genetic testing were given additional questions addressing outcome of their genetic testing and disclosure of results. In addition, they completed a 12‐item Likert scale series that assessed personal experience and feelings toward the testing process and outcome. Each Likert item contained a 10‐point scale, with responses ranging from “strongly agree” to “strongly disagree.” For analysis, responses were collapsed into three categories; agree, neutral, and disagree.

Data analysis was performed using JMP Version 11 software (SAS Institute, Cary, NC, USA). Associations between groups and binary characteristics were studied using proportions and Fisher's exact test. A two‐tailed *p*‐value of .05 or less was considered significant.

## 3 RESULTS

A positive family history of ALS (fALS) was reported by 45 of 449 (8.5%) of respondents; 341 of 449 (75.9%) reported a negative family history of ALS (sALS); and 63 of 449 (14.0%) indicated they did not know their family history status for ALS. Among 449 survey respondents who completed the portion of the survey addressing personal experience with genetic testing, 156 (34.7%) were offered testing, and 105 of 156 (67.3%) completed testing. The majority of fALS respondents (31/45, 68.9%) were offered testing, while a minority of sALS respondents (111/404, 27.5%) were offered testing (*p* = .00001). Approximately half of respondents with a positive family history of ALS (25/45, 55.6%) underwent genetic testing; 79 of 404 (19.5%) of all sALS respondents underwent testing (*p* = .05). A minority of respondents (12.5%) indicated contact with a genetic counselor, and these respondents were much more likely to be offered genetic testing (*p* = .00001) than those who did not indicate contact with a genetic counselor. Respondents with fALS were more likely to have seen a genetic counselor than respondents with sALS (*p* = .0082).

The test outcome (positive, negative, or inconclusive) was recalled and reported by 75 respondents (see Table 1). Respondents indicated that results were disclosed by a physician (69.4%), a genetic counselor (25.9%), “other” provider (8.2%), nurse (5.9%), and/or nurse practitioner (4.7%); multiple selections were permitted. Results disclosure took place during an office visit (65.1%), by letter (37.4%), or via phone (16.9%).

**Table 1:** Respondents who were offered and completed genetic testing (*n*;%)

	Genetic testing offered	Genetic testing completed	Recalled result
Yes	156; 34.7%	105; 67.3%	Positive result: 21; 28.0%
No	263; 58.6%	42; 26.9%	Negative result: 51; 68.0%
Don't know/remember	30; 6.7%	9; 5.8%	Inconclusive result: 3; 4.0%
*n*	449	156	75

Respondents who underwent genetic testing were asked to complete a 12‐item Likert series assessing their personal experience with testing. The majority of respondents agreed with each of 12 positive statements indicating satisfaction with pretest discussion, autonomy of test decision, results disclosure, explanation of results, emotional support, discussion of implications, and utility of results for the respondent and their family members. Specifically, most respondents indicated that genetic testing results were useful to them (70.8%; *n* = 68) and their families (62.5%; *n* = 60) with 83.3% (*n* = 80) agreeing that other persons with ALS should consider genetic testing (see Table 2).

**Table 2:** Respondent experience with ALS genetic testing

Question	Agree	Neutral	Disagree	Does not apply
The genetics of ALS was explained in a way that I could understand	88.5%	7.3%	4.2%	0.0%
I received the information I needed to make an informed decision about genetic testing	89.6%	6.2%	4.2%	0.0%
It was my decision to have genetic testing	90.7%	5.2%	3.1%	1.0%
I was satisfied with the way my test result was told/disclosed to me	75.0%	5.2%	8.3%	11.5%
My test result was explained to me in a way that I could understand	77.1%	7.3%	4.2%	11.5%
My questions about my test result were answered	71.9%	8.3%	5.2%	14.6%
My doctor/care team was emotionally supportive during the testing process	70.8%	17.7%	4.2%	7.3%
My doctor/care team explained what my result means for my children/family members	59.0%	15.8%	8.4%	16.8%
The results of my genetic testing were useful to me	70.8%	10.4%	7.3%	11.5%
The results of my genetic testing were useful to my family members	62.5%	18.8%	5.2%	13.5%
If I could “do it all over again,” I would choose to have genetic testing	80.1%	5.3%	4.2%	10.5%
I would recommend that other persons with ALS consider genetic testing	83.3%	12.5%	1.1%	3.1%

For analysis, responses were collapsed into three categories: “agree,” “neutral,” or “disagree”.

An overall mean test experience score was calculated for each respondent. Only six respondents (6/97, 6.2%) had negative or neutral mean test experience scores. Rank correlation analysis of individual respondents who completed at least two Likert scale items (*n* = 96) revealed that respondents who answered negatively to one item were also very likely to answer negatively to other items, confirming that a small group of respondents had a generally negative genetic testing experience. Of these six respondents, none knew the outcome of their testing: Four reported that they were not informed of their result, one indicated that they did not know their result and did not know if they were told, and one was informed of the result but could not recall it.

Comparison of mean Likert scale scores between groups revealed that respondents with fALS or sALS reported similar experiences with genetic testing, including those sALS respondents who tested positive despite no family history (*p* = .51). Respondents who indicated contact with a genetic counselor had a similar test experience as those respondents who did not indicate contact with a genetic counselor (*p* = .14). In addition, no differences in test experience scores were observed between those who received positive or negative genetic test results (*p* = .98).

## 4 DISCUSSION

The majority of respondents who underwent ALS genetic testing reported a positive experience and found value in testing. Most indicated satisfaction with each experience parameter queried, including items covering pretest discussion, autonomy of test decision, results disclosure, explanation of results, emotional support, discussion of implications, and utility of results for the respondent and their family members. Only six of 97 (6.2%) of respondents had negative or neutral mean test experience scores. Each of these six reported that they were not told or could not recall the outcome of their testing, suggesting that receipt and recollection of results is a necessary component of a positive genetic testing experience. The majority of respondents indicated receiving results during an office visit, suggesting a traditional face‐to‐face results disclosure with a clinician such as a neurologist or genetic counselor may in fact support a positive test experience, although specific methods (i.e., length of session, type of session) of results disclosure were not directly compared in this survey.

Mean test experience scores of respondents with a family history of ALS were not statistically different than those without a family history, indicating that sALS patients were equally satisfied with genetic testing as fALS patients. This satisfaction score also includes the few respondents (5) who tested positive for a mutation despite no family history of the disease. Although the proportion of sALS cases attributable to identifiable, highly penetrant mutations is increasing with continued gene discovery, the practice of offering genetic testing to sALS patients remains inconsistent. The European Federation of Neurological Societies (EFNS) issued guidelines indicating that genetic testing should not be performed in “cases with sporadic ALS with a typical classical ALS phenotype” (Andersen et al., 2012), while US guidelines do not address the issue (Miller et al., 2009). Concern about perceived patient anxiety for sALS patients undergoing genetic testing has been cited as a reason to refrain from offering testing to this population (Talbot 2014; Turner et al., 2017). Evidence suggests that most neurologists offer genetic testing to fALS patients, but the practice of offering it to sALS patients is more variable (Arthur et al., 2016; Byrne et al., 2012; Vajda et al., 2017). Our data indicate that sALS patients who did undergo testing found it useful and had a positive experience, which represents a factor to consider in the approach to the offer of testing.

We also examined whether seeing a genetic counselor was associated with a more positive test experience. Survey data collected on ALS patient attitudes indicated that patients who reported contact with a genetic counselor had more positive attitudes regarding the potential benefits of genetic testing for patients, the medical community, and society (Wagner et al., 2016). However, we found that seeing a genetic counselor was not associated with more positive test experience scores in our survey. The importance of genetic counseling in ALS, particularly in the context of genetic testing, has been emphasized (Chio et al., 2014; Fong, Karydas, & Goldman, 2012; Roggenbuck et al., 2017), but whether this is ideally provided by a neurologist or genetic counselor, or both, has not been addressed. Arthur et al. (2016) reported that 97.7% of neurologists indicate that they provide genetic counseling to ALS patients. Our data suggest that ALS patients who undergo genetic testing have a positive experience whether or not they see a genetic counselor.

Respondents who tested positive for an ALS mutation did not have significantly lower test experience scores, indicating that “getting bad news” did not negatively impact their perception of the testing process. This finding appears to be in concordance with the published literature suggesting that increased post‐test distress in persons undergoing genetic testing is usually transient and not clinically significant (Lerman, Croyle, Tercyak, & Hamann, 2002) Of note, all respondents who underwent testing and received a positive genetic test result chose “agree” or “strongly agree” to the Likert items stating “for me, the pros of genetic testing outweigh the cons” and “for society, the pros of genetic testing outweigh the cons.”

The lowest test satisfaction scores were observed in items related to implications for family members, including “My doctor/care team explained what my result means for my children/family members,” and “the results of my genetic testing were useful to my family members.” More thorough discussion of the implications of test results for family members may be beneficial for ALS patients and their families, as genetic testing results have implications for family members and may result in psychological and emotional effects for the extended family (Clarimon & Kulisevsky, 2013).

### 4.1 Study limitations

The study survey was sent to ATSDR registrants via a one‐time email announcement per CDC protocol. Previous survey research utilizing the registry for survey distribution reported a similar response rate of 11.5% (Malek et al., 2014). Study data may be biased if the response rate of this survey was not representative of the general ALS population. Although respondent demographic and disease characteristics mirror those of the national ALS population (Mehta et al., 2014), other respondent characteristics may not be representative. Most ATSDR registrants are enrolled from certified MDA or ALSA clinics, but most ALS patients do not receive care in such clinics (Mehta et al., 2014). Our results could therefore reflect an ascertainment bias skewed toward patients receiving specialized multidisciplinary care, actively engaged with research, and potentially more receptive to new technologies and care options. If our survey had reached a broader spectrum of ALS patients seen in diverse care settings, patient experience with and attitudes toward genetic testing may have been less positive.

Responses relied on patient recall of discussions by healthcare providers. We had no way of objectively assessing the quality of information that respondents received during the course of genetic testing and genetic counseling. As ALS patients may rely solely or primarily on the healthcare team for information, it seems possible that they “don't know what they don't know” and therefore may tend report satisfaction with poor and/or incomplete information. This could have biased test experiences in the positive direction.

Finally, numerous univariate tests were performed without correction for family‐wise error rate, and some statistically significant findings may be due to chance alone. However, due to the exploratory nature of this research, a less stringent *p*‐value was utilized during data analysis.

### 4.2 Practice implications

The finding that patients with both fALS and sALS report satisfaction and find value in genetic testing supports the practice of offering testing to all ALS patients. Patient‐reported outcomes are increasingly recognized as a critical component in shaping medical management policy (Stefanou & Amygdalos, 2015). In addition, seeing a genetic counselor was not associated with more positive test experience scores, suggesting that lack of access to a genetic counselor needs not be a barrier to a positive genetic testing experience (although the survey was not designed to measure or compare clinician competencies). It was noted that those respondents with neutral or negative mean test experience scores did not receive or recollect their test results, indicating that successful communication of test outcome is necessary aspect of a positive test experience. Finally, the lowest test experience scores were observed on items related to implications of results for family members, suggesting that patients could benefit from more extensive discussion of the complex issues surrounding transmission, penetrance, and testing of family members.

## 5 CONCLUSIONS

Current US best practice guidelines for ALS care do not provide recommendations for the offer of genetic testing. Available data suggest that neurologists are likely to offer genetic testing to patients with familial ALS, but the offer of genetic testing to patients with sporadic ALS is inconsistent. Our survey of a national ALS registry indicates that ALS patients find value in genetic testing, whether or not they have a family history of the condition. Genetic testing may help ALS patients to understand the cause of their condition, allow more accurate risk assessment and testing of family members, and facilitate clinical trial inclusion, including genotype‐specific treatments. As the genetic basis of ALS is further elucidated, genetic testing and counseling will become an increasingly vital component of ALS care.

## DISCLOSURES

Authors Karin N. Wagner, Dr. Haikady Nagaraja, Dawn C. Allain, Dr. Adam Quick, Dr. Stephen Kolb, and Jennifer Roggenbuck report no disclosures.
