# Supplementary material for: Patients with sporadic and familial amyotrophic lateral sclerosis found value in genetic testing
Source: Mol Genet Genomic Med. 2017 Dec 20;6(2):224–9. doi: 10.1002/mgg3.360 (PMC5902388; doi:10.1002/mgg3.360)
Supplement: Supplementary file 1 [file MGG3-6-224-s001.pdf]

SURVEY PROTOCOL
TITLE: Amyotrophic Lateral Sclerosis and Genetic Testing: A Perspective from the ALS Community
PRIMARY INVESTIGATOR: Jennifer Roggenbuck, MS LGC
(PLEASE NOTE: all *italicized* are for skip logic and/or notes for survey track)

Thank you for participating in this research survey. We hope that the result of this study will help us provide improved care and education for persons with ALS.

If you are unable to complete the survey on your own, a family member and/or caregiver may answer on your behalf. Participation in this research survey is voluntary and a refusal to participate will involve no penalty or loss of benefits to which you are otherwise entitled.

The survey will take about 20 minutes to complete. Please answer carefully because you may not go back to previous questions. The survey is not saved until it is completed.

You may terminate the survey at any time by closing your browser window. You may return to the survey later, but if you do so you will retake the survey from the beginning.

Once completed, the first 50 participants are eligible to enter a drawing to receive a $10 gift card to Starbucks (instructions for entry will be given upon survey completion) as compensation for your time and participation.

We will work to make sure that no one sees your survey responses without approval. But, because we are using the Internet, there is a chance that someone could access your online responses without permission. In some cases, this information could be used to identify you.

If you have any questions or concerns regarding this research study and/or your rights as a participant, please contact us at alssurveyosu@gmail.com or contact OSU ALS survey personnel (614) 688-7838.

THE OHIO STATE UNIVERSITY Medical Center

1. Are you affected with ALS (amyotrophic lateral sclerosis; aka "Lou Gehrig's Disease")?
    a. Yes, limb onset (started in arm or leg)
    b. Yes, bulbar onset (started with trouble speaking/swallowing)
    c. Yes, not sure which type
    d. No *(end survey)*
2. What best describes your race?
    a. White/Caucasian
    b. Black/African-American
    c. Asian
    d. Multiracial
    e. Other
    f. Prefer not to say
3. What is your age now?
    a. 39 or under
    b. 40 - 49
    c. 50 - 59
    d. 60 - 69
    e. 70 or over
4. What was your age at the time of your diagnosis?
    a. 39 or under
    b. 40 - 49
    c. 50 - 59
    d. 60 - 69
    e. 70 or over
5. When did you receive your diagnosis?
    a. 0 – 6 months ago
    b. 7 – 12 months ago
    c. 13 – 18 months ago
    d. 19 – 24 months ago
    e. 25 – 30 months ago
    f. 31 – 36 months ago
    g. More than 3 years ago
6. Do you have a family history of ALS? (choose all that apply)
    a. Yes, in my mother
    b. Yes, in my father
    c. Yes, in my child
    d. Yes, in my aunt or uncle
    e. Yes, in my grandparent
    f. Yes, in another relative(s)
    g. I do not know if I have a positive family history of ALS
    h. No
7. Do you have a family history of fronto-temporal dementia, Alzheimer disease, or other dementia? (choose all that apply)
    a. Yes, in my mother
    b. Yes, in my father
    c. Yes, in my child

    d. Yes, in my aunt or uncle
    e. Yes, in my grandparent
    f. Yes, in another relative(s)
    g. I do not know if I have a family history of these conditions
    h. No
8. Do you have a family history of Parkinson disease? (choose all that apply)
    a. Yes, in my mother
    b. Yes, in my father
    c. Yes, in my child
    d. Yes, in my aunt or uncle
    e. Yes, in my grandparent
    f. Yes, in another relative(s)
    g. I do not know if I have a family history of this condition
    h. No
9. Is your ALS doctor part of an ALS clinic?
    a. Yes, MDA - affiliated
    b. Yes, ALSA - affiliated
    c. Yes, both MDA and ALSA - affiliated
    d. Not sure
    e. No
10. Which specialists have you seen in your ALS clinic? (choose all that apply)
    a. Physical or Occupational Therapist
    b. Respiratory therapist
    c. Speech therapist
    d. Genetic Counselor
    e. Social Worker
    f. MDA or ALSA representative
11. Based on your knowledge or opinion, is ALS genetic (does it run in families)?
    a. Never
    b. Not usually
    c. Sometimes
    d. Most of the time
    e. Always
    f. Don't know
12. Based on your knowledge or opinion, is genetic testing for ALS available?
    a. Never
    b. Not usually
    c. Sometimes
    d. Most of the time
    e. Always
    f. Don't know
13. Based on your knowledge or opinion, is genetic testing available for family members of persons with ALS?
    a. Never
    b. Not usually
    c. Sometimes
    d. Most of the time
    e. Always

f. Don’t know

14. At your clinic, was the genetics or heredity of ALS discussed with you?
    a. No, this was not mentioned or discussed
    b. Yes, this was mentioned briefly
    c. Yes, this was explained and discussed with me
    d. Don’t remember
15. Did your doctor, or other care team member, tell you how often ALS is familial (runs in families)?
    a. No *(skip to question 17)*
    b. Yes
    c. Don’t know/don’t remember *(skip to question 17)*
16. Based on what your doctor/team member told you, how often is ALS familial (runs in the family)?
    a. Never
    b. Not usually
    c. Rarely
    d. Sometimes
    e. Often
    f. Always
17. Did you personally request information about genetic testing for ALS?
    a. Yes
    b. No
    c. Don’t know/Don’t remember
18. Was genetic testing for ALS offered to you?
    a. No *(skip to question 21)*
    b. Yes, only clinical testing was offered
    c. Yes, only research testing was offered
    d. Yes, clinical and research testing were offered
    e. Yes, I don’t remember which type was offered
    f. Don’t know/don’t remember *(skip to question 21)*
19. If genetic testing was offered, did you have genetic testing?
    a. No
    b. Yes *(skip to question 24)*
    c. Not sure
20. Why did you decide against genetic testing? (choose all that apply)
    a. Cost
    b. Did not believe that ALS is running in the family
    c. Do not wish to know if something is wrong
    d. Concerns about ability to obtain insurance
    e. Concerns about burdening my family members
21. Did your doctor/team member tell you what the chance is that your children could develop ALS?
    a. Yes, the chance was described as a percent *(skip to question 22)*
    b. Yes, the chance was described in words *(skip to question 23)*
    c. No *(skip to question 43)*
    d. Don’t know/don’t remember *(skip to question 43)*
22. Based on what your doctor/team member told you, what is the chance that your children could develop ALS? (please choose the range that includes the percent chance that was mentioned) *(skip to question 43 – Leikert scale)*

a. 0%
b. 1 - 4%
c. 5 - 9%
d. 10 - 24%
e. 25 - 49%
f. 50 - 74%
g. 75% or higher

23. Based on what your doctor/team member told you, what is the chance your children could develop ALS? *(skip to question 43 – Leikert scale)*
    a. Zero
    b. Very Low
    c. Low
    d. Moderate
    e. High
    f. Very High
24. Was your genetic testing done as a clinical test, or part of a research project?
    a. Clinical
    b. Research
    c. Not sure
25. Were you informed of the result of your genetic testing?
    a. No *(skip to question 35)*
    b. Yes
    c. Don't know/Don't remember *(skip to question 35)*
26. Who discussed your result with you? (choose all that apply)
    a. Doctor
    b. Nurse practitioner
    c. Nurse
    d. Genetic counselor
    e. Other
27. How were the results of genetic testing disclosed and explained to you (choose all that apply)?
    a. Telephone
    b. Office visit
    c. Letter
    d. Results were not given to me *(skip to question 34)*
28. What was the result of your genetic test?
    a. No gene or mutation was found *(skip to question 36)*
    b. The test was inconclusive *(skip to question 36)*
    c. Yes, a mutation or gene for ALS was found
    d. Don't know/don't remember *(skip to question 34)*
29. My test result identified a mutation in the following gene:
    a. C9orf72
    b. SOD1
    c. FUS
    d. TARDP
    e. Other
    f. Don't know/Don't remember
30. Did your doctor/team member tell you what the chance is that your children could inherit this gene?

a. No
b. Yes, the chance was described in words *(skip to question 32)*
c. Yes, the chance was described as a percent *(skip to question 33)*
d. Don't know/Don't remember

31. Based on your knowledge or opinion, what is the chance that your children would inherit the gene? *(skip to question 34)*
    a. 0%
    b. 25%
    c. 50%
    d. 75%
    e. 100%
    f. Other chance
    g. Don't know/Don't remember
32. Based on what your doctor/team member told you, what is the chance that your children would inherit this gene? *(skip to question 34)*
    a. Zero
    b. Low
    c. Moderate
    d. Very High
    e. Other
    f. Don't know/Don't remember
33. Based on what your doctor/team member told you, what is the chance that your children would inherit the gene?
    a. 0%
    b. 25%
    c. 50%
    d. 75%
    e. 100%
    f. Other chance
    g. Don't know/Don't remember
34. Did your doctor/team member discuss the chance that a gene carrier will actually develop ALS?
    a. No, this was not discussed
    b. Yes, they said that gene carriers always develop ALS *(skip to question 40)*
    c. Yes, they said that gene carriers usually develop ALS *(skip to question 40)*
    d. Yes, they said that gene carriers sometimes develop ALS *(skip to question 40)*
    e. Don't know/don't remember if this was discussed *(skip to question 40)*
35. Based on your knowledge or opinion, what is the chance that a carrier of an ALS gene will develop ALS? *(skip to question 39)*
    a. Gene carriers always develop ALS
    b. Gene carriers usually develop ALS
    c. Gene carriers sometimes develop ALS
    d. Gene carriers rarely develop ALS
    e. Don't know
36. Did you doctor/team member discuss the chance that your children could develop ALS?
    a. No *(skip to question 39)*
    b. Yes, the chance was described in words *(skip to question 38)*
    c. Yes, the chance was described as a percent
    d. Don't know/don't remember *(skip to question 39)*

37. Based on what your doctor/team member told you, what is the chance that your children could develop ALS? *(skip to question 42)*
    a. 0%
    b. 1 - 4%
    c. 5 - 9%
    d. 10 - 24%
    e. 25 - 49%
    f. 50 - 74%
    g. 75% or higher
38. Based on what your doctor/team member told you, what is the chance your children could develop ALS? *(skip to question 42)*
    a. Zero
    b. Very Low
    c. Low
    d. Moderate
    e. High
    f. Very High
39. Based on your knowledge or opinion, what is the chance your children could develop ALS? *(skip to question 42)*
    a. Zero
    b. Very Low
    c. Low
    d. Moderate
    e. High
    f. Very High
40. Did your doctor/team member say that genetic testing could be offered for your unaffected family members?
    a. No
    b. Yes
    c. Don't know/Don't remember
41. Did any of your family members have genetic testing for ALS? (choose all that apply)
    a. No
    b. Yes, family member(s) with ALS
    c. Yes, family member(s) who do not have ALS
    d. Don't know
42. Based on YOUR EXPERIENCE with genetic testing for ALS, please indicate whether you agree with the following statements: *(Leikert scale)*
    1 - Strongly Agree
    2 - Agree
    3 - Somewhat agree
    4 - Maybe agree
    5 - Neutral
    6 - Maybe disagree
    7 - Somewhat disagree
    8 - Disagree
    9 - Strongly disagree
    0 - Does not apply
    a. The genetics of ALS was explained in a way that I could understand

b. I received the information I needed to make an informed decision about genetic testing
c. It was my decision to have genetic testing
d. I was satisfied with the way my test result was told/disclosed to me
e. My test result was explained to me in a way I could understand
f. My questions about my test result were answered
g. My doctor/care team was emotionally supportive during the testing process
h. My doctor/care team explained what my result means for my children/family members
i. The results of my genetic testing were useful to me
j. The results of my genetic testing were useful to my family members
k. If I could 'do it over again' I would choose to have genetic testing for ALS
l. I would recommend that other persons with ALS consider genetic testing

43. Based on your knowledge or opinion, please indicate whether you agree or disagree with the following statements*: (Leikert scale)*

1 - Strongly Agree
2 - Agree
3 - Somewhat agree
4 - Maybe agree
5 - Neutral
6 - Maybe disagree
7 - Somewhat disagree
8 - Disagree
9 - Strongly disagree
0 - Does not apply

a. Genetic Testing should be offered to patients with ALS
b. Genetic Testing should be offered to patients with ALS who have a family history of ALS
c. Genetic testing can provide useful information to patients with ALS
d. Genetic testing should be offered to family members of persons with ALS
e. Genetic testing should be offered to family members of persons with ALS who have a gene/mutation
f. Genetic testing can provide useful information to family members of persons with ALS
g. If I were offered genetic testing for ALS, I would have genetic testing
h. If my adult children wanted genetic testing for ALS, I would support them
i. Genetic testing can help doctors understand my condition
j. In the future, genetic testing may help doctors treat ALS
k. For me, the pros of genetic testing for ALS outweigh the cons
l. For society, the pros of genetic testing for ALS outweigh the cons

Thank-you for completing this survey.

If you would like more information about genetic testing options for ALS, please talk to your doctor and/or visit these resources:
Amyotrophic Lateral Sclerosis Association at ALSA.org
Muscular Dystrophy Association at MDA.org
National Society of Genetic Counseling at www.nsgc.org (click "find a counselor")

To enter for the $10 gift card to Starbucks, please send your email address to: ALSsurveyOSU@gmail.com. The first 50 email submissions will be selected for the gift card and you will be contacted by research personnel for a mailing address to distribute your winnings. You may withdraw your information at any time. No personal emails, addresses or other identifiers will be published and all participation for this drawing is voluntary. Once winnings are distributed, all personal information provided will be eliminated from the study, and the ALSsurveyOSU@gmail.com account deactivated from further use.
